# Autoimmunity and Cancer—Two Sides of the Same Coin

**DOI:** 10.3389/fimmu.2022.793234

**Published:** 2022-05-13

**Authors:** Justyna Sakowska, Łukasz Arcimowicz, Martyna Jankowiak, Ines Papak, Aleksandra Markiewicz, Katarzyna Dziubek, Małgorzata Kurkowiak, Sachin Kote, Karolina Kaźmierczak-Siedlecka, Karol Połom, Natalia Marek-Trzonkowska, Piotr Trzonkowski

**Affiliations:** ^1^ Department of Medical Immunology, Medical University of Gdańsk, Gdańsk, Poland; ^2^ International Centre for Cancer Vaccine Science, University of Gdańsk, Gdańsk, Poland; ^3^ Laboratory of Translational Oncology, Intercollegiate Faculty of Biotechnology, University of Gdańsk and Medical University of Gdańsk, Gdańsk, Poland; ^4^ Department of Surgical Oncology, Medical University of Gdańsk, Gdańsk, Poland; ^5^ Laboratory of Immunoregulation and Cellular Therapies, Department of Family Medicine, Medical University of Gdańsk, Gdańsk, Poland

**Keywords:** immune tolerance, autoimmune diseases, cancer immunology, tumor microenvironment, regulatory cells

## Abstract

Autoimmune disease results from the immune response against self-antigens, while cancer develops when the immune system does not respond to malignant cells. Thus, for years, autoimmunity and cancer have been considered as two separate fields of research that do not have a lot in common. However, the discovery of immune checkpoints and the development of anti-cancer drugs targeting PD-1 (programmed cell death receptor 1) and CTLA-4 (cytotoxic T lymphocyte antigen 4) pathways proved that studying autoimmune diseases can be extremely helpful in the development of novel anti-cancer drugs. Therefore, autoimmunity and cancer seem to be just two sides of the same coin. In the current review, we broadly discuss how various regulatory cell populations, effector molecules, genetic predisposition, and environmental factors contribute to the loss of self-tolerance in autoimmunity or tolerance induction to cancer. With the current paper, we also aim to convince the readers that the pathways involved in cancer and autoimmune disease development consist of similar molecular players working in opposite directions. Therefore, a deep understanding of the two sides of immune tolerance is crucial for the proper designing of novel and selective immunotherapies.

## Introduction

Immune tolerance is a state of unresponsiveness of the immune system to self-tissues with a concomitant ability to identify and respond against non-self and dangerous antigens. Multiple mechanisms shape and control this state, including the elimination of autoreactive receptors from the system in bone marrow and the thymus (central tolerance). However, not all autoreactive cells are deleted in the primary lymphoid organs. For example, the naive T-cell repertoire that leaves the thymus contains up to 40% of low-avidity self-reactive T cells. These cells can potentially trigger an autoimmune response; therefore, several mechanisms of peripheral tolerance evolved to prevent their activation (1). Specialized cell subsets, such as regulatory T (Tregs) and B cells (Bregs), tolerogenic dendritic cells (tolDCs), and M2 macrophages, participate in keeping the balance between tolerance and activation. However, genetic predispositions and epigenetic modifications combined with exposure to environmental factors can disrupt this status, resulting in the development of autoimmunity. Therefore, an increasing number of approaches that boost the immune tolerance have been evaluated and were already implemented for the treatment of autoimmune diseases in humans. On the other hand, the same mechanisms can be exploited by cancer to set up cancer tolerance (2). In fact, the attraction of tolerogenic cell subsets and evading immune response is considered as one of the hallmarks of cancer. The malignant cells used to express immune checkpoint proteins show impaired antigen presentation, undergo epithelial-to-mesenchymal transition (EMT), or present alterations in RNA editing. In consequence, the presence of a tumor-specific antigen (TSA) or tumor-associated antigen (TAA) does not elicit immune responses to malignant cells (3). Therefore, multiple approaches have been already made to break cancer tolerance and awaken the immune system for the fight against cancer. These strategies were based on monoclonal antibodies, adoptive cell therapies, or therapeutic anti-cancer vaccines. Nevertheless, there is still a lack of full understanding of the complex network of mechanisms leading to tolerance induction or its breakdown. Therefore, with the current review, we aim to discuss the mechanisms involved in the development of autoimmunity and cancer, shedding a light simultaneously on two sides of the same coin. We hope that our paper will sort out the current knowledge in the field and inspire future studies on immune tolerance.

## Microbiome and Immune Response

Gut microbiota imbalance is associated with the development and progression of multiple diseases, such as gastrointestinal cancers or inflammatory bowel disease. The link between gut dysbiosis and tumor development has been already reported with *Helicobacter pylori* being the best studied pathogen in this context (4, 5). However, this is definitely not the only component of the digestive tract microbiome involved in carcinogenesis. However, not only the composition of microbiota but also its activity have an impact on cancer development. Microbial metabolites, such as short-chain fatty acids (SCAFs) or N-nitroso compounds (NOCs) showed anti- and procarcinogenic effects, respectively (6, 7). The microbiome, as well as its metabolites, also affects the function of the immune system and, in this way, may contribute to cancer tolerance or the stimulation of anti-cancer responses. For instance, the fungal genus *Candida*, which is detected in 74% of oral cancer patients, was reported to increase the proliferation of myeloid-derived suppressor cells (MDSCs) known to dampen the anti-cancer response (8). Therefore, not surprisingly, gut microbiota may affect the efficacy of anti-cancer management as it was reported for immunotherapy with immune checkpoint inhibitors. For instance, the abundance of *Bifidobacterium* species or *Akkermansia muciniphila* (next-generation probiotic bacteria) was associated with slow tumor growth and beneficial responses to anti-PD-1 (programmed death receptor 1) therapy (9–11). Therefore, the modulation of gut microbiota may positively affect treatment efficiency and thus patient survival.

On the other hand, the interactions between immunological, microbial, and environmental factors in genetically susceptible individuals are involved in the etiopathogenesis of Crohn’s disease (12, 13). Dysbiotic microbial alterations, such as low gut microbiota diversity, as well as a decreased amount of bacteria belonging to the *Firmicutes* phylum, are observed in patients with Crohn’s disease (14). The link between mutations in TLR4 (Toll-like receptor 4) (rs4986790) and the IL-10 receptor with *Mycobacterium avium* subspecies paratuberculosis in these patients was also noted (15).

The nucleotide-binding oligomerization domain-containing protein 2/caspase recruitment domain-containing protein 15 *(NOD2/CARD15)* gene located on chromosome 16q12 was the first described gene connected with Crohn’s disease pathogenesis (16, 17). It encodes the NOD2 protein, which is mainly expressed not only by dendritic cells (DCs) and monocytes but also enterocytes and Paneth cells. The molecule is known to play a significant role in the intestinal innate immune response against the bacterial cell wall. More than 30 variants of the *NOD2/CARD15* gene have been identified, while an increased risk of Crohn’s disease development was connected to R702W, G908R, and L1007fs variants, as well as P268S and IVS8+158 polymorphisms (17).

The role of microorganisms in autoimmunity development was also extensively studied for type 1 diabetes (T1D). Molecular mimicry is described as the structural similarity between self- and foreign (microbial) antigens and has been connected with the break of tolerance to pancreatic beta cells in T1D (18). Researchers described a number of homologies between the antigens of beta cells and microorganisms such as Coxsackievirus (19) or Rotaviruses (20). These data demonstrate a big dynamism of the immune status and suggest that tuning the microbial repertoire may skew the immune response to the desirable profile to fight the cancer or restore immune tolerance to self-antigens.

## Escape From Central Tolerance Mechanisms and Cancer Immune Evasion

There is a considerable body of literature presenting the different genetic factors that are associated with specific disease phenotypes as well as with the risk of the disease occurrence (21). Various alleles of human leukocyte antigen (HLA) class I and class II molecules were reported to be associated with a particular autoimmune disease occurrence, including T1D, multiple sclerosis (MS), rheumatoid arthritis (RA), or celiac disease (22, 23). The exact mechanism of how HLA polymorphisms predispose to autoimmunity remains poorly understood. However, it is suggested that differences in the binding affinity of HLA molecules to autoantigens might be involved (24). Nevertheless, the association between autoimmune disorders and the polymorphisms of other genes involved in immune cell antigen recognition and activation like protein tyrosine phosphatase non-receptor type 22 (PTPN22), cytokines, chemokine receptors, costimulatory molecules, and inhibitory checkpoints were also identified (25).

The hallmark of autoimmunity is the presence of autoreactive T and B cells that were not deleted by the mechanisms of central tolerance (26). One of the most studied defects of T-cell-negative selection is mutations in the transcriptional autoimmune regulator gene (*AIRE*). *AIRE* is mainly expressed by the thymic medullary epithelial cells (mTECs) and is responsible for the expression of tissue-restricted antigens within the thymus. The T cells responding to these antigens are considered self-reactive and eliminated through negative selection. Thus, when *AIRE* is defective, the T cells specific to self-antigens leave the thymus and enter circulation. This results in a variety of autoimmune disorders (27, 28). The mouse models of *Aire* knockout showed that the *AIRE* expression prevents multiorgan lymphocyte infiltration, various organ-specific autoantibodies, and infertility (29). In humans, *AIRE* mutations lead to a severe condition called autoimmune polyendocrinopathy syndrome type 1 (APS1) (30, 31). In addition, it was observed that *AIRE* expression is regulated by sex hormones, leading to sexual dimorphism in autoimmune diseases (32, 33). For example, the castration of male animals led to a lower thymic expression of AIRE, while estrogen treatment resulted in the downregulation of AIRE in cultured human thymic epithelial cells (TECs). In addition, AIRE levels in the human thymus grafted into immunodeficient mice differed according to the sex of the recipient (32, 33). Therefore, AIRE has also been extensively studied in the context of reproductive system cancers. Kalra et al. reported that the AIRE expression in prostate cancer is responsible for resistance to anti-cancer therapy and increased invasiveness. AIRE^+^ prostate cancer cells were shown to secrete increased levels of IL-6 and prostaglandin 2 (PGE2), which polarized the tumor-associated macrophage toward the M2 phenotype with an increased expression of CD206 and CD163 antigens. In addition, prostate cancer growth and lymphadenopathy after subcutaneous tumor engraftment were only observed in the AIRE^+/+^ animal model. On the contrary, AIRE^-/-^ mice showed small benign tumors (33).

The defects of the central tolerance mechanism of B cells, observed in a number of autoimmune diseases, result in the accumulation of autoreactive B cells in the periphery. The mutations of PTPN22, Bruton’s tyrosine kinase (BTK), adenosine deaminase (ADA), impaired BCR light-chain rearrangements, and Toll-like receptor (TLR) alterations were observed to contribute to the increase in autoreactive B cells (34). Recently, PTPN22 also emerged as a potential target for cancer immunotherapy. It is not surprising as PTPN22 plays an inhibitory role in the antigen-specific responses of both T and B cells; dectin-1 signaling in DCs; the development and function of Tregs; the macrophage functions mediated *via* TLRs, NOD2, and NLRP3; and neutrophil adherence and mast cell activation in an IgE-dependent manner (35). Several single-nucleotide polymorphisms in the *PTPN22* gene were identified. The most extensively studied is a missense mutation at position 1858 (C3T), resulting in the substitution of an Arg (R) at position 620 to Trp (W). The generation of the Lyp620W variant (also identified as rs2476601) of the protein was found to impair the negative selection of autoreactive T and B cells during their development in the thymus and bone marrow, respectively, and the generation of self-reactive antibodies (36, 37). In consequence, the Lyp620W variant of PTPN22 was identified in multiple autoimmune diseases, including T1D, RA, systemic lupus erythematosus (SLE), Graves’ disease, and myasthenia gravis (38–41). On the other hand, the same variant of PTPN22 was reported to augment antitumor responses and be associated with lower cancer incidence (35, 42). For example, the carriers of the *PTPN22(C1858T)* variant have a lower risk of non-melanoma skin cancer, while the homozygotes for the *PTPN*22*(C1858T)* have improved survival when treated with atezolizumab (anti-PDL1 antibody). These data underline again that immune tolerance is indispensable for preventing autoimmunity, but lowering the threshold of T-cell activation can improve tumor control and the efficacy of anti-cancer treatment.

Cancer immune evasion and autoimmunity prevalence can also be affected by sex hormones. Differences in the male and female endocrine systems lead to discrepancies in the quality and quantity of their immune responses. It was reported that while the female immune system provides better antimicrobial and anticancer responses, it is also more prone to autoimmune diseases (43). Estrogen levels are higher during pregnancy and are correlated with an increased proportion of Tregs in peripheral blood (44). Accordingly, the incidence of relapses of MS in pregnant women decreases significantly (45). Both innate and adaptive immune cells express estrogen receptors α and β (higher expression was observed in B cells than T cells, NK cells, and monocytes) that activate protolerogenic effects (46). Estrogens drive the polarization of T cells into Th2 and Treg cells; increase the production of IL-4, IL-10, and transforming growth factor-β (TGF-β); induce the expression of GATA-3, FoxP3, PD-1, and CTLA-4 (cytotoxic T-lymphocyte antigen 4) on T cells; and reduce the Tfh (T follicular helper cell) response (47, 48). On the other hand, SLE patients experience more flares during pregnancy (49). Interestingly, B-cell tolerance is regulated by estrogens at the maturation stage by engaging estrogen receptor α. Estradiol was shown to be responsible for decreased B-cell lymphopoiesis while expanding the population of splenic marginal-zone B cells through the increase of BAFF concentration (50). Estrogens were also shown to influence immune cells in the tumor microenvironment (TME). Certain mutations in the estrogen receptor result in an increase of tumor-infiltrating Tregs and T helper cells (51). It was also reported that estrogens influence tumor-associated macrophages, directing their polarization into the M2 phenotype and thus promoting their immunosuppressive activity (52, 53).

## Immune Checkpoints

Immune checkpoints are inhibitory receptors that convey negative signals to immune cells, preventing autoimmunity (54). The importance of immune checkpoints in supporting tolerance and preventing autoimmunity development is best observed in knockout mice models. For instance, the lack of CTLA-4, PD-1, BTLA (B- and T-lymphocyte attenuator), TIGIT (T-cell immunoreceptor with immunoglobulin and ITIM domain), and VISTA (V-domain Ig suppressor of T-cell activation) was shown to cause massive lymphoproliferation, an onset of autoimmune diseases, or fatal multiorgan tissue destruction (notably CTLA-4 deficiency) (55–61). In humans, several polymorphisms of immune checkpoint genes were identified and reported to be associated with susceptibility to autoimmune diseases (62–70).

CTLA-4 is a critical regulator of T-cell responses expressed by Tregs and activated conventional T cells. The main role of the receptor is to inhibit antigen presentation and the following activation of naive T cells by competitive binding to costimulatory receptors CD80 and CD86 on antigen-presenting cells (APCs) (71, 72). It was reported that CTLA-4 not only binds its ligands but also captures and removes them from APCs by a process of trans-endocytosis. In consequence, these costimulatory molecules are degraded inside CTLA-4-expressing cells resulting in a temporary lack of CD80/CD86 on APCs and thus impaired costimulation *via* CD28 (73). CTLA-4 is indispensable for preventing autoreactivity (74, 75). Its deficiency in humans is a common hallmark of primary immune deficiencies associated with immune dysregulation and prominent autoimmunity with highly variable features. The clinical symptoms probably result from the aberrant activation of polyclonal T cells. In addition, the deficiency of CTLA-4 results in increased CD28 co-stimulation that triggers self-reactive T cells against a variety of tissues. Treg dysfunction plays a vast role in the immune activation associated with CTLA-4 loss-of-function mutations (75). On the contrary, CTLA-4 expression on tumor cells was recognized as a prognostic factor of poor outcome in breast, pancreatic, and nasopharyngeal cancers (76–78). The application of therapeutic antibodies targeting CTLA-4 such as ipilimumab became a breakthrough in cancer therapy. Anti-CTLA-4 antibodies were shown to unlock the immune response to cancer, as well as lead to the depletion of tumor-infiltrating Tregs *via* antibody-dependent cell-mediated cytotoxicity. This way, anti-CTLA-4 demonstrated durable clinical activity in a subset of patients with solid malignancies including advanced melanoma (79–81).

Programmed cell death receptor 1 (PD-1) is another immune checkpoint significant for self-tolerance and the cessation of the immune response that became a target of cancer immunotherapy. Upon engagement by its ligand (PD-L1, Programmed cell death ligand 1), PD-1 acts as a brake to the immune system that induces the apoptosis of activated T cells (82). PD-L1 expression can be detected in pancreatic islets, vascular endothelial cells, and placenta where it is responsible for tissue protection from autoimmune responses (83). For example, in T1D, PD-L1 was observed to be upregulated in insulin-producing beta cells under an autoimmune attack and correlated with the intensity of CD8^+^ T-cell infiltration in the pancreas (84, 85). In addition, PD-1/PD-L1 interaction was reported to be involved in the generation of inducible Tregs (iTregs). Francisco et al. showed that PD-L1-negative APCs had an impaired ability to generate Tregs, either *in vitro* or *in vivo* (86). The failure of APCs isolated from SLE patients to upregulate PD-L1 expression validates these findings in humans (87). The blockade of PD-1 or PD-L1 in experimental models of autoimmunity led to disease onset and exacerbation (88, 89), indicating the essential role of these immune checkpoints in tolerance and, specifically, in Treg maintenance. Recent reports on autoimmune-related adverse events in oncologic patients treated with PD-1/PD-L1 axis blockers support these findings (90, 91).

In cancer, effector T cells, which are persistently exposed to antigen stimulation in TME, express PD-1 at high levels, in the long term, causing T-cell functional exhaustion. It results in the inability of T cells to eliminate tumor cells and facilitates cancer progression (34, 92). Additionally, cancer cells actively exploit PD-L1 to evade the immune system and hijack the immunosurveillance mechanisms with PD-L1 expression (93). Moreover, the results presented by Chen et al. (2018) revealed that apart from cell surface expression, PD-L1 was present in extracellular vesicles (exosomes) produced by melanoma cells, suggesting its systematic immunosuppressive impact (94). As a result, it leads to the transcriptomic changes and the exhaustion of CD4^+^ (95) and CD8^+^ (96) T cells that are unable to eliminate cancer cells effectively. In a vast number of cancers, lymphocyte infiltration is in positive correlation with PD-L1 expression, which is simply an adaptive mechanism of the tumor to escape an immune response. Even though tumor PD-L1 expression usually suggests poor prognosis, then higher levels of tumor PD-L1 expression correlate with a better efficiency of immunotherapy (97).

Another molecule involved in central and peripheral tolerance is Fas. Fas/FasL ligation on TCR-stimulated lymphocytes restricts the overactivation of immune cells after an antigenic challenge, called activation-induced cell death (AICD). It is one of the main mechanisms in restoring immune homeostasis (98). The Fas/FasL-induced apoptosis of B cells was shown to be important in germinal center reactions (98). FasL can be expressed on non-immune cells in immune-privileged sites such as the eye, brain, and placenta, restricting the access of activated immune cells to these tissues (99). Alterations in Fas-mediated apoptosis were implicated in the pathogenesis of autoimmune diseases. Mutations in Fas/FasL axis-related genes lead to a striking lymphoproliferation with autoimmune cytopenias in humans termed autoimmune lymphoproliferative syndrome (ALPS) (100, 101). An interesting feature of ALPS is an accumulation of double-negative T cells that are terminally differentiated, with the markers of immune exhaustion (102). On the other hand, increased expression of FasL was observed in T1D (103), autoimmune thyroid diseases (104), and in MS (105, 106). An interesting feature of Fas/FasL signaling is the opposite outcome of ligation with membrane-bound versus soluble forms of these molecules where the soluble Fas and FasL do not induce apoptosis (107, 108). This discovery prompted studies investigating the levels of serum Fas/FasL molecules in autoimmune diseases, revealing elevated levels in SLE patients (107, 109) and Sjögren’s syndrome (SS) (110). Excessive Fas signaling in the tumor microenvironment, majorly caused by high levels of the Fas ligand released by myeloid-derived suppressor cells (MDSCs), leads to the apoptosis of tumor-infiltrating lymphocytes (TILs) and was described as one of the core reasons for the failure of cancer immunotherapy (111). In addition, FasL was reported to be expressed in numerous cancer types with a potential to induce the apoptosis of immune cells in the TME and was associated with poor prognosis. On the other hand, there is still controversy when it comes to the role of Fas/FasL axis in cancer cells. Several *in vitro* studies suggest that the ultimate effect may depend on the level of FasL expression by tumor cells. As elevated levels of FasL cause neutrophil-mediated inflammation that leads to tumor rejection, surprisingly low levels of FasL seemed to facilitate tumor growth. The Fas/FasL role in cancer is still not fully understood and brings a lot of controversies but surely requires further investigation as targeting Fas may significantly improve the efficiency of immunotherapy and tumor rejection (112, 113).

Other known immune checkpoints include BTLA, T-cell immunoglobulin and mucin domain-3 (TIM-3), and TIGIT (114, 115). In general, all were shown to inhibit the responses of activated T cells, while BTLA also demonstrated an impact on B cells (116). It was observed that patients with SLE and MS present a low expression of BTLA on B and T cells (117–119). Its decreased expression on naïve B cells was associated with increased IFN-γ and autoantibody levels in SLE patients that could suggest alterations in B-cell activation during the course of the disease (118). In conditions where Th17/Treg balance is shifted, the involvement of immune checkpoint signaling pathways was also implicated. A study by Wu et al. described a lower frequency of TIM-3 positive T cells together with increased IL-17 levels in patients suffering from autoimmune hepatitis, and experiments on mice confirmed that the blockade of TIM-3 signaling aggravated liver injury (120). TIGIT has been recently associated with Treg biology through the transcriptional profiling of these cells. It was suggested to be a marker of natural thymus-derived Tregs (tTregs) with strong suppressive activity and lineage stability (121). It competes with the CD226 molecule for binding a costimulatory poliovirus receptor (PVR) CD155 and inhibitory CD112 (Nectin-2) expressed on DCs (121). TIGIT-CD226 signaling in T cells was shown to be implicated in the pathogenesis of experimental autoimmune encephalomyelitis (EAE). CD226 knockout EAE mice showed favorable Th17/Treg proportion and increased TIGIT and CTLA-4 expression on Tregs (122). On the other hand, the lack of TIGIT resulted in increased levels of proinflammatory cytokines and hindered IL-10 production by T cells (61). Recently, a novel ligand for TIGIT was discovered on cancer cells. Nectin4 was reported to bind exclusively to the TIGIT molecule (123). TIGIT-Nectin4 interaction inhibits natural killer (NK) cell activity, which is a crucial element of the anti-cancer immune response. In addition, antibodies blocking Nectin4 induced enhancement of tumor killing in vitro and *in vivo* (123).

## Regulatory T Cells

Central tolerance is crucial for the development of a small subset of intermediate-affinity, self-reactive T-cell clones that are rescued from deletion and become (tTregs) (124, 125). Apart from tTregs, Tregs can be induced on the periphery from naïve or effector T cells, becoming peripheral Tregs (pTregs). In addition, specific Treg subpopulations can be distinguished based on secreted cytokines, such as type 1 regulatory T cells (Tr1), T-helper type 3 cells (Th3), and IL-35-producing regulatory T cells (iTr35). They secrete IL-10, TGF-β, and IL-35, respectively (126–128). Functionally, follicular Tregs (Tfr) can also be distinguished within the FoxP3^+^ population (129). Tfr cells have a TCR repertoire resembling tTregs and were shown to be able to control germinal center reactions and antibody production (130, 131).

Tregs exert their immune-suppressive effects using diverse mechanisms. The most important are (1) a high expression of immune checkpoint inhibitors; (2) infectious tolerance, where Tregs exert and transfer suppressive activity toward other immune cells when activated by autoantigens (132); (3) the secretion of anti-inflammatory cytokines (133), (4) IL-2 deprivation, and (5) adenosine accumulation *via* CD39 and CD73 activities (134). Apart from cytokines, extracellular vesicles are recently gaining attention as a way of efficient intercellular communication with a significant role in the regulation of the immune system (135, 136).

**Figure 1:**
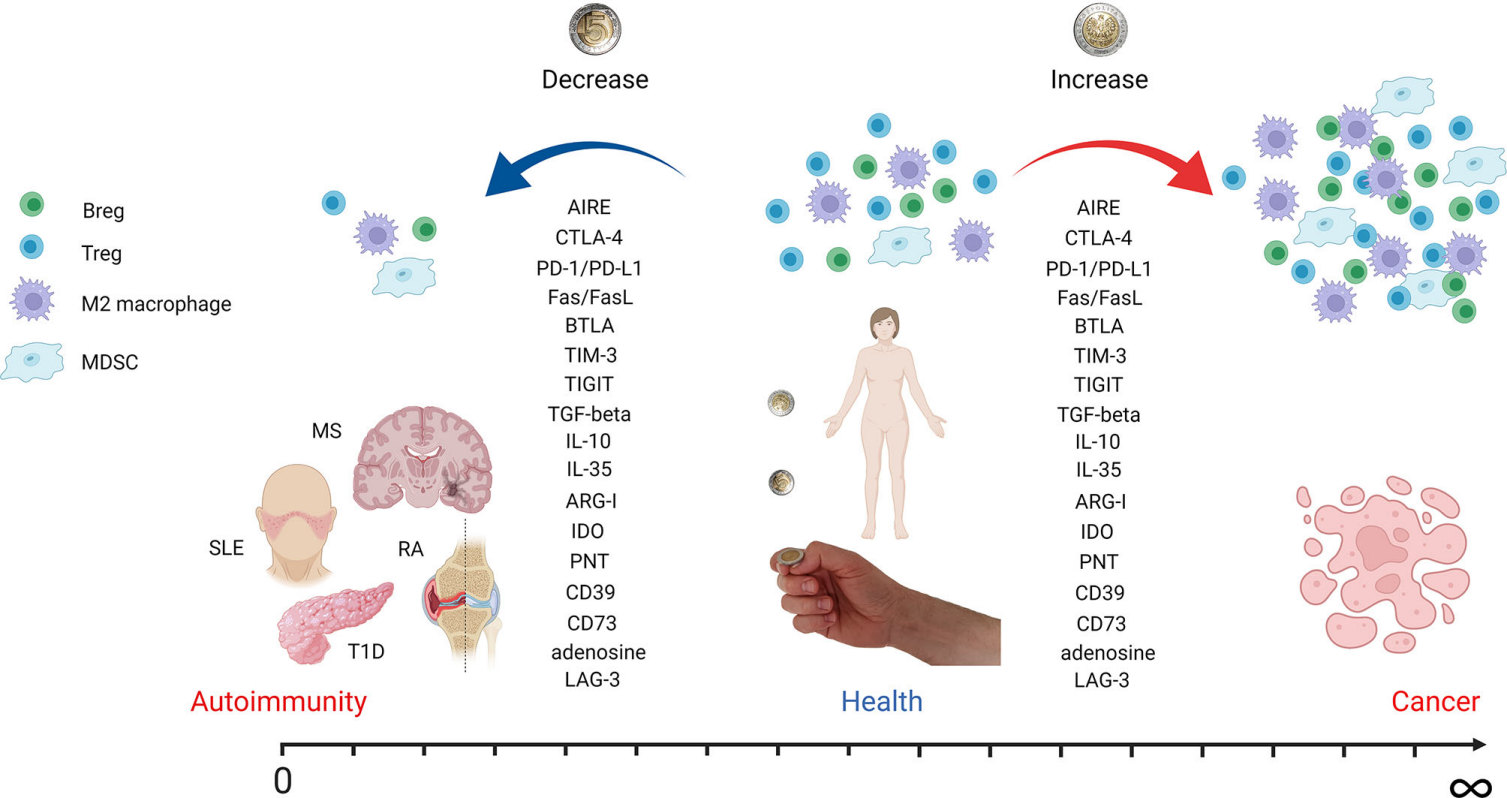
Autoimmunity and cancer as two sides of the same coin. The figure depicts how tuning of immune system regulatory mechanisms can contribute to autoimmunity, health, or cancer development. A decrease in regulatory cell populations like Tregs, Bregs, M2 macrophages, and MDSCs leads to autoimmune disease onset. However, an increase in the same cell subsets is associated with cancer development and progression. Effector molecules involved in immune tolerance induction are downregulated in autoimmunity but overexpressed in cancer. The most important molecules mentioned in the text are listed. AIRE, autoimmune regulator; CTLA-4, cytotoxic T-lymphocyte antigen 4; PD-1, programmed cell death receptor 1; PD-L1, programmed cell death ligand 1; BTLA, B- and T-lymphocyte attenuator; TIM-3, T-cell immunoreceptor with immunoglobulin and ITIM domain; TIGIT, T-cell immunoglobulin and ITIM domain; TGF-beta, transforming growth factor beta; IL, interleukin; ARG-I, arginase I; IDO, indoleamine-pyrrole 2,3-dioxygenase; PNT, peroxynitrite; LAG-3, lymphocyte-activation gene 3 (figure created with BioRender.com).

Tregs are crucial for preventing autoimmune reactions (**Figure 1**). They play an important role in immune tolerance maintenance, as their deficiency causes immune dysregulation, polyendocrinopathy, enteropathy, and X-linked (IPEX) syndrome, leading to multiorgan autoimmune damage when not treated (137, 138). Numerous studies described quantitative Treg changes in autoimmune diseases. A decrease in the Treg population was shown in juvenile idiopathic arthritis (139) and RA (140). However, in some diseases, such as systemic sclerosis (SSc), Tregs were shown to be increased (141). The results from SLE patients regarding Treg frequencies are conflicting, which may arise from differences in the analyzed phenotypes of Tregs (142). Numerous studies suggested the decreased immunosuppressive potential of Tregs in autoimmune diseases (143–147). The main limitation of studying Tregs in human organ-specific diseases is usually the lack of insight into the damaged tissue, as systemic and local immune responses may differ dramatically. Nevertheless, several studies pursued this problem. For instance, Marazuela et al. reported lower numbers of Tr1 and higher proportions of tTregs in the thyroid glands of patients with autoimmune thyroid disease (AITD) as compared with peripheral blood (145, 146). In patients with relapsing–remitting MS (RR-MS), higher frequencies of Tregs were present in cerebrospinal fluid (CSF) rather than in the peripheral blood. The same group of patients had decreased peripheral blood Treg levels compared to the patients with secondary-progressing MS and other neurological diseases, suggesting the migration of Tregs to the site of autoimmune inflammation (148). In addition, the primary role of tTregs, as opposed to pTregs, was demonstrated to control T1D development. However, the deficiency in pTregs increased the incidence of insulitis (149). In the synovial fluid of arthritis patients, high frequencies of iTregs and tTregs were present; however, tTregs presented an unstable FoxP3 expression. Moreover, FoxP3^-^ Tregs were converted to IL-17-producing cells under the environment of the inflamed joint (150, 151). The Th17 cytokine profile (IL-17, IL-12, IFN-γ) influences the organ tissue environment, causing chronic inflammation and, ultimately, organ failure (152). Considering the close transcriptional programs of Th17 and Tregs, both depending on TGF-β, Tregs in the presence of IL-6 were shown to be converted into Th17 cells, or IL-17+ ex-regulatory T cells (exTregs). This plasticity of Tregs results in the blunting of suppressive capacity and the secretion of proinflammatory IL-17 and IFN-γ (153–155). On the other hand, cytokines IL-10 and TGF-β enable the differentiation of immune cells into anti-inflammatory Tregs, Bregs, tolDCs, and M2 macrophages (155).

Indisputably, within the TME, Tregs are present in high frequencies. Treg presence is accommodated by the immunosuppressive cytokine milieu at the site as well as the chemotactic factors produced in TME. High numbers of FoxP3^+^-expressing Tregs infiltrating TME in lung, breast, and pancreatic cancers were associated with poor prognosis (156). Tregs express various chemokine receptors, like CCR4 and CCR5, that allow migration to TME more efficiently (157, 158). TME is rich in TGF-β and promotes the differentiation of conventional CD4^+^ T cells into pTregs (159). Resting Tregs are not immunosuppressive unless they become activated through TCR engagement and signaling molecules. The Tregs found in TME are, however, highly activated and immunosuppressive, characterized by upregulated levels of the master regulatory transcription factor FoxP3 (160). This subsequently leads to the suppression of CD8^+^ T cells, NK cells, NKT cells, and M1 macrophages and the maturation of DCs through IL-10, TGF-β, and indoleamine-pyrrole 2,3-dioxygenase (IDO) (161–163). In addition, Tregs not only bind IL-2 competitively to conventional T cells but also release soluble CD25 (IL-2R subunit) that eliminates IL-2 and alters cytotoxic T-cell functions. Tregs in TME may also release IL-35 that increases the expression of inhibitory receptors like PD1, TIM-3, and lymphocyte-activation gene 3 (LAG-3). This leads to the exhaustion of TILs (164–166). Interestingly, Treg elimination that was followed by cancer antigen vaccination generated effective anti-tumor CD4^+^ and CD8^+^ T-cell responses in cancer patients with advanced malignancies (167). However, as mentioned before, systemic Treg depletion would lead to severe autoimmune disorders, emphasizing the need for more selective methods that would specifically target intratumoral Tregs.

## Regulatory B Cells

B-cell maturation mechanisms require consecutive checkpoints to develop tolerance: clonal deletion, receptor editing, and anergy. Immature B cells transmitting an overly strong signal through the B-cell receptor (BCR) in response to self-antigen undergo clonal deletion. A tolerance mechanism unique to B cells is the possibility of repeated immunoglobulin light-chain gene recombination. Such rearrangements lead to alterations in BCR specificity to ideally avoid the formation of self-reactive B-cell clones (168, 169). The subsets of B cells expressing PD-1 (170), TIM-3 (171), and BTLA (117) were described as Bregs, an important element for the maintenance of peripheral tolerance (**Figure 1**). However, a consensus regarding the definition and detailed phenotype of Bregs has not yet been reached. The distinct methods for identification in various disease models and different tissues complicate the general classification. IL-10, TGF-β, and IL-35 have been identified as the main suppressive cytokines produced by Bregs; thus, some authors used to classify the cells into IL-10^+^, TGF-β^+^, and IL-35^+^ Bregs (172). Among IL-10^+^ human Bregs, the following phenotypes of Bregs were reported: CD1d^hi^ CD5^+^ (173), CD5^+^ (174), CD24^hi^CD27^+^ (175), CD24^hi^CD38^hi^ (176–178), CD25^+^CD71^+^CD73^−^, and CD25^+^CD71^+^CD73^low^PD-L1^+^ (179), CD154^+^ (180), CD5^hi^CD38^low^PD-1^hi^ (181), CD27^int^CD38^+^ (182). Up to now, 2 subsets of TGF-β^+^ Bregs have been identified in humans: CD25^hi^CD27^hi^CD86^hi^ CD1d^hi^ (183) and CD24^hi^CD38^hi^ (178). Despite the fact that IL35^+^ B cells have been identified in humans, up to now, specific surface markers have not been reported for these cells in men (172, 184). The manipulation of the Breg compartment through the adoptive transfer of isolated or *ex vivo*-induced cells was explored in the murine models of autoimmune diseases. For example, IL-10^+^ Bregs were shown to suppress inflammation in the mice models of RA, EAE, and SLE. The most prominent therapeutic effects were observed when Bregs were administered early in the disease course (183, 185–188). The mechanisms used by Bregs have not been studied extensively. Nevertheless, *in vitro* studies performed by Kessel et al. resulted in several interesting observations. Human Bregs defined as CD25^high^ CD27^high^ CD86^high^ CD1d^high^ IL-10^high^ TGF-β^high^ cells were shown to significantly decrease the proliferation of autologous conventional CD4^+^ T cells in a dose-dependent manner. In addition, Bregs were found to upregulate FoxP3 and CTLA-4 expression in Tregs in cell-to-cell-dependent contact. The effect was even stronger when Bregs were pretreated with a TLR-9 agonist (oligodeoxynucleotide) and CD40L (183). The other groups also reported the suppressive effects of Bregs on DC and macrophage cytokine production and antigen presentation (175, 189). Increased frequencies of IL-10^+^ B cells and their progenitors were found in patients with various autoimmune diseases, such as SLE, RA, SS, autoimmune vesiculobullous skin disease, and MS. However, the significance of Bregs in the pathogenesis of human autoimmune diseases is yet to be determined (175).

The impact of B cells in cancer is still unclear and ambiguous as they were shown to play a role in both cancer promotion and anti-cancer responses (190). Significant B-cell infiltration was found in breast cancer, non-small cell lung cancer (NSCLC), ovarian cancer, melanoma, and renal cell carcinoma. Bregs have been also identified in a number of cancers including lung (191), gastric (192), and breast cancers (193). Increased infiltration with Bregs results in the inhibition of effector T-cell responses and their impaired proliferation. It was suggested that the tumor and TME can direct tumor-infiltrating B cells into tumor-induced Bregs (tBregs) (194) by the direct tumor cell: B-cell contact (195). Lindner et al. reported that tumor-infiltrating Bregs use Granzyme B for the degradation of the CD3 ζ-chain in CD4^+^ T cells. The phenomenon results in a limited proliferation of the target CD4^+^ T cells (196). Interestingly, tBregs were also shown to play a substantial role in the education of MDSCs, enhancing cancer-induced immune suppression (197). In addition, Breg-derived IL-10 leads to the conversion of conventional B cells into Bregs and contributes to Treg expansion (183). tBregs were also found to direct conventional CD4^+^ T cells into Tregs in breast and gastric cancers (177, 198). Another study utilizing a mouse model showed that tumor-educated Bregs suppress not only the proliferation of helper and cytotoxic T cells but also the secretion of Th-1 cytokines and the expansion of NK cells in a TGF-β- or PD-L1-dependent manner (195). A similar immunosuppressive activity was reported for IL-35^+^ Bregs. Breg-derived IL-35 was shown to stimulate cancer (199), as well as convert both T and B cells into Tregs and Bregs, respectively. Several surface molecules have been identified to be involved in direct cell-to-cell interactions between Bregs and the target immune cells, like Bregs CD40/CD40L, CTLA-4/CD80 and CD86, PD-L1/PD-1, or Fas/FasL (200–203).

## Myeloid-Derived Suppressor Cells

A significant population of cells identified within the tumor was described as activated immature myeloid cells with immunosuppressive function, termed myeloid-derived suppressor cells (MDSCs). These cells, in general, can be divided into 2 populations: mononuclear (M-MDSCs; CD11b^+^Ly6G^−^Ly6C^hi^) and polymorphonuclear/granulocytic MDSCs (PMN-MDSCs; CD11b^+^Ly6G^+/hi^Ly6C^low/int^) (204). The granulocyte monocyte-colony stimulating factor (GM-CSF), vascular endothelial growth factor (VEGF), stem cell factor (SCF), prostaglandins, TNF-α, IFN-γ, and IL-18 were shown to promote the differentiation of functional MDSCs that contributed to the establishment of immunosuppressive niche and tumor progression (205–209). MDSCs were shown to be engaged in the suppression of TIL activity, EMT, and angiogenesis and participate in establishing a pre-metastatic niche (210, 211). In addition, the increased production of nitric oxide (NO) by MDSCs resulting from inducible nitric oxide synthase (iNOS) overexpression was reported to be responsible for T-cell apoptosis and proliferation suppression, as well as the inhibition of antigen presentation by DCs (212, 213). Moreover, MDSCs isolated from tumor-bearing animals showed significantly higher levels of reactive oxygen species (ROS) than the cells isolated from healthy controls. Further studies demonstrated that ROS are crucial for the MDSC suppression of T-cell proliferation, survival, and TCR signaling (214–216). It was also reported that MDSCs express elevated levels of arginase I (ARG-I; **Figure 1**). This way, they can deplete TME from indispensable amino acids, such as L-arginine or cysteine affecting T-cell activation and proliferation (217, 218). One of the mechanisms that stands behind this T-cell suppression is the downregulation of the CD3 ζ-chain of the TCR complex (219). Tumor-derived MDSCs are also a potent source of IDO, an L-tryptophan-degrading enzyme that induces the suppression of T-cell proliferation and survival, as well as promotes Treg induction (220–222). Another important effector molecule used by MDSCs is peroxynitrite (PNT). The production of PNT in TME was shown to nitrate the TCR complex, leading to the unresponsiveness of tumor-infiltrating cytotoxic T lymphocytes to the specific antigens presented by MDSCs (223–225).

It is also recognized that MDSCs participate in the generation of immunosuppressive adenosine (226, 227). MDSCs express the ectoenzymes triphosphate diphosphohydrolase 1 (NTPDase 1/CD39) and ecto-5’-nucleotidase (5’-NT/CD73). The first ectoenzyme is responsible for the hydrolysis of extracellular ATP or ADP into AMP, which is then degraded by CD73 into adenosine. Adenosine is known to inhibit the activation and effector function of T cells, mainly by A2A and A3 adenosine receptors (228). However, these receptors can also be found at the surface of MDSCs. The blockade of the A2B receptor was shown to reduce the secretion of IL-10 and monocyte chemoattractant protein 1 (MCP-1) by MDSCs in mice with melanoma (229). Aside from IL-10, TGF-β is another cytokine important for MDSC function. MDSC-derived IL-10 and TGF-β promote the differentiation of T cells into Tregs and suppress T- and NK-cell activation as well as DC function (230, 231). TGF-β was reported to induce EMT in cancer cells (211, 232), generate pro-tumorigenic M2 macrophages (233), and drive pro-tumorigenic neutrophil polarization (234). In NSCLC, higher levels of TGF-β were associated with an increased expression of inhibitory molecules such as CTLA-4 and TIM3 on cancer cells (235). It was suggested that MDSCs are responsible for the induction and recruitment of the Treg population in the TME. While the process of Treg induction is not fully elucidated and was suggested to depend on cytokine milieu and cell-to-cell contact, the Treg recruitment was shown to be largely dependent on the production of CCL2 and CCL5 chemokines (236, 237). On the other hand, MDSCs may also limit the T-cell infiltration of the tumor by metalloproteinase 17 (ADAM17), which cleaves L-selectin (CD62L) present on the surface of naïve T cells. In consequence, T cells are not able to infiltrate tumor or enter peripheral lymph nodes (238).

The growing body of research on MDSCs and their suppressive capacity in TME sparked interest for the exploration of their role and potential therapeutic use in autoimmune diseases. In the aim to diminish the heterogeneity of studied MDSCs, they used to be divided into M-MDSC (CD11b^+^Ly6G^−^Ly6C^hi^) and PMN-MDSC (CD11b^+^Ly6G^+/hi^Ly6C^low/int^) subsets as in cancer studies (239, 240). Multiple studies on the animal model of MS have pointed to the beneficial role of MDSCs in autoimmunity. Moliné-Velázquez et al. identified ARG-I positive MDSCs in the spinal cord during the course of EAE. The cells showed tropism to demyelinated areas in CNS. The density of ARG-I^+^ MDSC infiltrate, as well as the local proportion of the apoptotic T cells, correlated with the disease course and clinical state. They peaked in parallel with the clinical score, which were decreased significantly during remission, and was not detectable in the chronic phase (240). These data correspond with the previous studies that reported the presence of ARG-I^+^ cells exclusively when the switch from proinflammatory to anti-inflammatory conditions occurred and the active phase was about to end (241–243). These data indicate that MDSCs are involved in limiting inflammatory damage in MS and contribute to relative recovery in the remitting phase of the disease.

In humans, as in previously described animal studies, the numbers of MDSCs were found to be an indicator of the disease phase. For example, RR-MS was characterized by significantly higher levels of the PMN-MDSC subset in the peripheral blood at relapse than in the remission period or in healthy individuals. Experiments *in vitro* revealed that PMN-MDSCs from patients with RR-MS suppress autologous T-cell proliferation, suggesting their beneficial role for remission induction (244).

However, higher proportions of M-MDSCs were observed to be positively correlated with proinflammatory Th17 and Th1 cells, as well as with a worsened metabolic profile in the patients with T1D and their relatives at elevated risk for the disease (245). Similar patterns were described in RA (246) and SLE (247). These data indicate that a detailed characterization of MDSC subsets and their further stratification is inevitable if MDSCs are planned to be harnessed to stop autoimmune diseases. Nonetheless, the idea of utilizing the suppressive activity of MDSCs in therapy prompted the experiments of adoptive transfer of MDSCs to diabetes-prone mice that successfully prevented the onset of autoimmune diabetes and established tolerance to self-antigens *via* Treg induction (248).

## Macrophages

Macrophages can be divided into two main groups, classically activated, proinflammatory macrophages (M1) and alternatively activated macrophages (M2) with anti-inflammatory and regenerative properties. M1 and M2 cells can be distinguished by secreted cytokines, for example, INF-γ, IL-1, IL-6, IL-12 and IL-10, and TGF-β, respectively. However, macrophages exhibit exceptional plasticity depending on the microenvironment (249). It has been reported that tumor-associated macrophages (TAMs) are recruited to TME by chemokines, such as CCL2 in different tumors, including glioblastoma and breast and lung cancers (250–252). Moreover, TAMs start to produce CCL2 and thus recruit more macrophages and stimulate their polarization toward a pro-tumoral M2 phenotype (253–256). Targeting TAMs in pancreatic ductal adenocarcinoma by inhibiting CCR2 has shown a therapeutic benefit by restoring anti-tumor immunity in preclinical models (257). Although TAMs can produce IL-8, a chemotactic factor for T cells, high levels of IL-8 in plasma, peripheral mononuclear cells, and TAMs were negatively correlated with clinical prognosis regardless of high CD8^+^ T-cell infiltration in the tumor (258). TAM-derived cytokines include IL-6, IL-10, and TGF-β. IL-6 combined with IL-6R can activate anti-apoptotic pathways in tumor cells and prolong their survival (259). A meta-analysis revealed that the serum levels of IL-10 are positively correlated with tumor progression, showing the importance of TAMs in the promotion of tumor development (**Figure 1**) (260). Additionally, TAMs secrete inflammatory mediators, including prostaglandin E2 (PGE2) and matrix metalloproteinase-7 (MMP-7). These molecules interfered with TLR-mediated or IFN-γ-mediated DC and macrophage activation. In addition, a direct induction of genes that suppress APC function was observed. Thus, TAMs indirectly impair the T-cell recognition of tumor antigens (261).

Macrophages are constantly present in peripheral tissues, where they can rapidly act as APC, as shown in the T1D animal model (262). In autoimmune diseases, the overreaction of the immune system and the resulting highly proinflammatory environment lead to tissue damage. Therefore, the imbalance in M1/M2 macrophage subsets was observed in several autoimmune diseases, both organ specific (MS) (263) and systemic with in-tissue manifestations (RA, SLE, SSc; **Figure 1**) (264). Recent studies on human pancreata from T1D patients, using multiparametric analyses, revealed the presence of macrophages of mixed M1/M2 characteristics, confirming the high plasticity of these cells (265, 266). Studies on EAE showed that the polarization of macrophages follows the natural pattern of the disease with the increase of M2 macrophages during the remission phase (263). The adoptive transfer of M2 macrophages in the mouse model of SLE decreased the disease severity score (267) and prevented diabetes in NOD mice (268). Importantly, these transferred cells were homed to the site of ongoing insulitis (268). These results suggest an attractive therapeutic opportunity.

## Fibroblasts

TME contains a special subpopulation of fibroblasts with a myofibroblastic phenotype. Cancer-associated fibroblasts (CAFs) are activated, but unlike in a physiological wound-healing process and tissue repair, they remain constantly activated, leading to pathological fibrosis. Active fibroblasts and myofibroblasts are the main effectors involved in the initiation of fibrosis due to excessive collagen deposition and the modulation of extracellular matrix (ECM) (269, 270). Multiple mechanisms can be involved in their activation, like the composition of the (ECM), DNA damage, physiological stress (mediated by ROS), inflammatory signals (e.g., IL-1 and IL-6), and growth factors, fibroblast growth factor (FGF) and platelet-derived growth factor (PDGF) (271–273). Once activated, they are sufficient not only to promote tumor growth but also to further model ECM; produce proinflammatory cytokines, proangiogenic VEGF, and the chemokine ligand CXCL12 that is responsible for attracting immunosuppressive cells into TME that indirectly assist in immune tolerance establishment” as this part of the sentence is continuation of the role of CXCL12 (274). It was reported that throughout the secretion of TGF-β, CAFs induce the occurrence of EMT and promote lung metastasis in breast cancer (275). Moreover, the cytokine is involved in the synthesis of collagen and matrix modification by macrophages and fibroblasts, leading to local tissue scarification, like pulmonary fibrosis (276). Tissue fibrosis and the contractile properties of myofibroblasts stiffen ECM subsequently, lowering blood circulation and leading to local tissue hypoxia (277). These effects also reduce the possibility of cytotoxic effectors to reach cancer cells, therefore reducing immune surveillance and therapy efficacy. While using the combinations of multiple biomarkers to help identify cell subsets in TME, it has been found that the presence of CAFs is negatively correlated with the prognosis in patients receiving PD-1 immunotherapy in metastatic melanoma (266). This shows that the combination of different biomarkers can not only help us target CAFs as a potential clinical marker for the success of therapy, but targeting CAFs can also improve the efficacy of immunotherapy. Inhibiting the growth and proliferation of CAFs and preventing or reversing their activation status are potential ways to target CAFs in cancer therapy.

The therapeutic application of fibroblasts in autoimmune diseases has not been extensively studied. Jalili et al. reported tolerance induction by fibroblasts in the animal model of T1D and pancreatic islet transplantation. However, the therapeutic fibroblasts were transduced with a lentiviral vector carrying IDO cDNA. Thus, the cells artificially overexpressed IDO and efficiently suppressed immune responses (278, 279) Nevertheless, the data of Khosravi-Maharlooei et al. suggest the potential therapeutic use of fibroblasts in autoimmune diseases. They showed that fibroblasts can condition DCs to express higher levels of co-inhibitory molecules and anti-inflammatory cytokines. In addition, fibroblasts arrested the ability of DCs to induce the proliferation of T cells in both direct and indirect pathways. Fibroblast-primed DCs were also reported to migrate to the regional lymph nodes and present fibroblast-derived antigens. This study sheds light on the role of fibroblasts in the maintenance of self-tolerance and regulation of immune responses (280). Finally, the data provide inspiration for the future therapeutic approaches.

## Epithelial-to-Mesenchymal Transition in Cancer and Autoimmune Disorders

Another complex phenomenon modulating immunity is EMT, which induces morphological changes in epithelial cells, after which, they start to resemble mesenchymal cells—fibroblasts (281–284). As a result, cells undergoing EMT show increased motility and invasiveness due to the degradation of extracellular matrix, but it can also acquire other features, like stem cell properties or the ability to escape the immune system, which overall contributes to the aggressive phenotype of cancers (281, 285, 286). A direct connection between immunotolerance and EMT was shown in breast and lung cancer *in vitro* studies, where upon EMT induction, the expression of PD-L1 in cancer cells increased (287, 288). Moreover, cells with a mesenchymal phenotype showed higher levels of PD-L1 than cells of epithelial phenotype (288). Hypoxic hepatoma cells, which undergo EMT, induce IDO expression in monocyte-derived macrophages and further suppress the proliferation of T cells as well as promote the expansion of Tregs (289). Pancreatic tumors with EMT features co-express PD-L1, and melanoma cells with EMT features show increased NK immunosuppressive function in comparison to epithelial melanomas (290), which overall indicates that EMT in cancer cells leads to a decreased immune response. On the other hand, the EMT inducers present in the tumor microenvironment can modify the activity and composition of the immune cells in the tumor niche. TGF-β, a potent inducer of EMT in multiple cancers, including breast (291, 292), lung (235, 293, 294), and colon (295, 296) cancers, exerts immunosuppressive function (235). In lung adenocarcinoma, the EMT signature of the tumor was associated with increased infiltration by CD4^+^ FoxP3^+^ Tregs (297), a decreased infiltration of activated effector T cells (including Th17 cells), and higher levels of activated B cells and γδ T-cells (235). Similarly, in patients with pancreatic ductal adenocarcinoma, tumors with mesenchymal features have decreased the number of CD8^+^ T cells and increased the frequencies of Tregs (298).

EMT develops in response to chronic inflammation where it can lead to pathological fibrosis-the generation of myofibroblasts, which actively deposit ECM, leading to a decreased functionality of the affected organs (299–301). The triggers for EMT and fibrosis are overlapping; most importantly, both require TGF-β (302, 303). Chronic inflammation in autoimmune disorders such as RA, Crohn’s disease, SLE, or scleroderma have been associated with fibrotic tissue remodeling (300, 304, 305). The local proinflammatory environment is not neutral for tissue-resident mesenchymal cells/fibroblasts that become activated and, as ECM-producing cells, exacerbate fibrosis. Signaling through the proinflammatory IL-17A receptor was responsible for fibroblast activation and the fibrosis of lung tissue in RA-associated lung disease and idiopathic pulmonary fibrosis (306). It seems that during chronic inflammation, overridden tolerance mechanisms interfere in the natural process of healing and repair mediated by fibroblasts, which can additionally support inflammation.

## RNA Editing

One of the mechanisms used by the innate immune response for self- vs. non-self-recognition is the RNA-editing process. There are two main types of RNA editing: (i) adenosine-to-inosine (A-to-I) conversion catalyzed by adenosine deaminases acting on RNA (ADAR) enzymes and (ii) cytidine to uridine (C-to-U) deamination by apolipoprotein B mRNA-editing catalytic polypeptide-like (APOBEC) family. A-to-I RNA editing allows cells to mark the host RNA as self. This way, the cell is able to recognize and tolerate edited self-RNAs with viral dsRNA sensors (such as PKR, MDA5, and RIG-I) and simultaneously discriminate non-edited dsRNAs present in the cells as viral genetic material (307). This launches an innate immune response, and results in death of cells where non-edited dsRNA was detected. Defects in RNA editing may contribute to autoimmune diseases and are observed in various cancers (308, 309).

The role of RNA editing and the enzymes involved in this process in cancer are currently being explored (308). Potentially, RNA editing may lead to presentation of edited and thus changed peptides by the MHC class I molecules of malignant cells. This phenomenon was recently shown in melanoma, where TILs were able to recognize the peptides derived from the ADAR1-edited form of cyclin I (CCNI) presented on the surface of cancer cells (310). These findings suggest that either the absence of or a higher expression of ADAR1 can result in novel ADAR1-dependent neoantigens that may be used as biomarkers in cancer or as potential targets for cancer immunotherapy. The study of Asaoka et al. supports this hypothesis. The increase of APOBEC3-mediated RNA editing in breast cancer was correlated with a higher T-cell infiltration of the tumor, improved survival, and better prognosis (311). The role of RNA editing in immune regulation is also proven by the fact that the expression of some RNA-editing enzymes is dependent on IFN (312). The knockdown of *Adar1* in mouse B16 melanoma cells was shown to increase the susceptibility of the tumor cells to anti-PD1 therapy after engraftment to animal model (313). Interestingly, *Adar1* knockout does not disturb growth of B16 cells in culture but mediates killing of B16 *Adar1* ^-/-^ cells by T lymphocytes *in vivo*. This effect is determined by abnormal activation of the intracellular dsRNA sensors (Mda5 and PKR) by unedited intracellular dsRNA mimicking virus infection (314). In contrary to B16 mouse melanoma cells, in many human cancer cell lines, loss of *ADAR1* results in cell death, even in the absence of innate immune cells. These ADAR1-dependent tumors usually show high IFN induction, probably through the innate immune DNA sensor STING (315) and have a higher expression of both: IFN-stimulated genes (ISGs, including *ADAR1*) and innate immune sensors for dsRNA, than other types of tumor cells. In addition, they are sensitive to elevated levels of dsRNAs while *ADAR1* knockdown is lethal for these cells through the Mda5/MAVS and PKR pathways (315).

RNA editing is also involved in autoimmune diseases connected to the dysregulation of IFN signaling. For instance, mutations in the *ADAR1* gene were identified to be involved in the development of type I interferonopathies, including Aicardi–Goutieres syndrome (316), dyschromatosis symmetrica hereditiaria (317), bilateral striatal necrosis (318), and spastic paraplegia (319). ADAR1 expression was shown to be also involved in RA or SLE (320, 321). The enzyme was over-expressed in synovium of RA patients regardless of the disease duration. In addition, the ADAR1p150 isoform was found to be elevated in the blood of the patients with active RA. Interestingly, decreased baseline ADAR1p150 expression and the individual adenosine RNA editing rate of cathepsin S AluSx^+^ in RA were indicators of a good clinical response to the treatment (320).

## Discussion

Immune response and tolerance are vital for proper reaction against pathogens and maintaining internal homeostasis. For years, immunologists have been studying the mechanisms’ underlying tolerance to fight autoimmune diseases. However, a deeper understanding of immune tolerance in TME as well as the mechanisms underlying autoimmunity may help to generate an antitumor response and break tolerance to cancer. Phenomena, such as the generation of tolerogenic immune cell populations or EMT, are revealing pathways that lead to immunological changes in the tumor milieu. Anti-cancer immunotherapies should attempt to break immune tolerance toward the tumor; otherwise, the efficacy of such treatments is greatly limited. On the other hand, the immunotherapies aiming to combat autoimmune diseases seek to induce immunological tolerance, therefore, to limit the pathological immune reaction against self-antigens. As potent tolerance to cancer and the lack of self-tolerance in autoimmune diseases stand on two sides of the same coin (**Figure 1**; **Table 1**), certain lessons can be learned from the understanding of these two fields of medicine. We believe that combining knowledge from research on autoimmune diseases and cancer therapies will lead to a considerable progress in both areas. The advantages of exchanging knowledge between these two research fields can already be observed in the therapeutic strategies that are being developed. For instance, while genetically engineered super-activated CAR T cells have been successfully applied for the therapy of non-solid malignancies (322), the depletion of autoreactive immune cells gives promising results in the treatment of autoimmune diseases (323, 324). Moreover, the therapeutic potential of CAR Tregs is being explored in the context of autoimmunity (325), as antigen-specific Tregs proved to have better control over autoreactive effector cells than polyclonal Tregs (326). The strategy has already proved its efficacy in the animal models of MS (327), colitis (328), and T1D (329). Another example of a similar therapeutic approach in cancer and autoimmune diseases are adoptive cellular therapies, such as those that use mature DCs in cancer and tolDCs in autoimmune diseases. In cancer research, DCs loaded with tumor antigens are used as a cancer vaccine (330). In the therapy of autoimmune diseases, tolDCs presenting synovial fluid-derived peptides have been recently tested in a phase I clinical trial in RA patients (331). Many of the immune regulatory axes can be targeted in both autoimmune diseases and cancer, usually in an opposite manner—targeting different cytokines (including IL-2, IL-6, IL-10, IL-15, IL-17, and TNF-α) to manipulate the tolerance and increasing or decreasing the regulatory populations of the cells. As presented in this review, cytokine imbalance is a vital component of TME or autoimmune disorders that creates an opportunity for therapeutic intervention. On the other hand, therapies depleting or promoting the expansion of effector subsets of immune cells are also valid therapeutical strategies, for example, the depletion of effector cells in autoimmune diseases and adoptive cell therapy in cancer patients (322).

**Table 1:** Mechanisms involved in breaking tolerance to self-tissues and in induction of cancer tolerance.

	Autoimmunity	Cancer-induced tolerance
General tolerance mechanism	Escape from central tolerance and impaired peripheral tolerance	Escape from immune recognition and induction of peripheral tolerance
Subsets of regulatory cells	↓ Function and/or quantity of tTregs and pTregs	↑ Tregs, induction of pTregs and Bregs in tumor microenvironment
	↑ MDSCs during active disease	↑ Suppressive activity of MDSCs in tumor microenvironment
Activity of cells	↓ Migration of regulatory cells	↑ Migration of regulatory cells
	↓ Immune checkpoint expression by immune cells	↑ Immune checkpoint expression by immune cells and tumor cells
Cytokines	↑ Proinflammatory cytokines	↑ Immunosuppressive cytokines
Chronic effects on immune cells	Differentiation of Tregs into inflammatory IL-17^+^ exTregs	Exhaustion of TILs

MDSC, myeloid-derived suppressor cell; Tregs, regulatory T cells; pTregs, peripheral Tregs; TIL, tumor-infiltrating lymphocyte; tTregs, thymus-derived Tregs; Bregs, B regulatory cells. ↓, decrease; ↑, increase.

Immune checkpoint inhibitors were found to be a milestone in cancer therapy. Ipilimumab, the first immune checkpoint-blocking antibody targeting CTLA-4, was approved by the FDA. It was used for the first time in 2002 and later approved in 2011 for treating unresectable melanoma (332). PD-1-inhibiting antibodies have been also successfully used for the treatment of multiple cancer types as they are at least partially able to reinvigorate exhausted T-cells that regain the cytotoxicity against the cancer (333). Mechanistically, PD-1 signaling acts as a brake to the immune system but it can be stopped by implementation of either PD-1 or PD-L1 blocking monoclonal antibodies that are able to directly inactivate the PD-L1 inhibitory signaling in TME, reverse T-cell exhaustion, and ultimately induce tumor regression (334, 335). Nivolumab, pembrolizumab, and cemiplimab are FDA-approved PD-1-blocking antibodies for the treatment of various cancers including melanoma, renal cell carcinoma, NSCLC, and squamous cell carcinoma. However, many other indications are waiting for the approval (336). When it comes to PD-L1 inhibitors, currently, FDA has approved the following three: atezolizumab, durvalumab, and avelumab (337). At the same time, immune checkpoint fusion proteins are arising as a tool in the treatment of autoimmune diseases. The first promising results of exploiting the inhibitory activity of CTLA-4 in animal models of autoimmune diseases were presented over 25 years ago (338, 339). Successful clinical trials in human patients with psoriasis vulgaris, RA, and juvenile idiopathic arthritis led to the FDA approval of abatacept in 2005 (340–342). CTLA-4Ig is also tested in MS (343) and T1D (344, 345); however, these organ-specific diseases were far less responsive to this therapeutic agent. Experimental studies revealed that CTLA-4Ig induced the suppression of tolDCs (346) and Treg differentiation (347), improved the Treg function (348), and decreased the numbers of Th2 cells (349). The fusion proteins of PD-1 also convey immunomodulatory properties (350). Consequently, other immune checkpoint fusion proteins or agonistic antibodies, such as TIGIT-Fc, TIGIT mAb, and VISTA mAb, are evaluated in pre-clinical and clinical trials (351–353).

A particularly attractive therapeutic approach is the generation of an antigen-specific response with antigen-based and cell-based anti-cancer vaccines (354). These type of vaccines also constitute an extensively investigated strategy to induce tolerance in autoimmune diseases (355). Noteworthy, the combined use of different therapeutic strategies proved to be a valid option for enhancing the response to therapy in both—cancer and autoimmune disease (356, 357). However, therapeutic strategies need to be focused on restoring balance in the immune system and be applied with caution, as the overstimulation of the immune system in cancer may lead to the development of autoimmune disorders (358, 359). On the other hand, over-suppression in the treatment of autoimmune diseases might create a window of opportunity for cancer growth and progression (360, 361).

We hope that with the current paper, we were able to give a glimpse into the mechanisms that regulate tolerance to self-tissues and cancer. A dynamic balance between the resting and activation states is crucial to keep the organism safe from external and internal threats like pathogenic microorganisms, cancer cells, or hypersensitivity. We believe that a better understanding of these mechanisms opens the opportunities for novel and selective immunotherapies.

## Author Contributions

JS, ŁA, MJ, NM-T, and PT contributed to the concept of the review. All authors wrote the manuscript. NM-T revised the manuscript with the contribution of all authors. All authors read and approved the manuscript.

## Funding

The study was funded by project ”International Centre for Cancer Vaccine Science” that is carried out within the International Research Agendas Programme of the Foundation for Polish Science co-financed by the European Union under the European Regional Development Fund (NM-T), National Science Centre, Poland (funding decision no. DEC-2011/01/D/NZ3/00262, granted to NM-T), and by internal grant of Medical University of Gdańsk ST-49 (PT). This work was supported by the National Science Centre grant number 2016/21/D/NZ3/02629 (AM). The publication of the article was supported by the project POWR.03.05.00-00-z082/18 (JS) cofinanced by the European Union through the European Social Fund under the Operational Programme Knowledge Education Development 2014–2020.

## Conflict of Interest

NM-T and PT are the co-authors of 2 patents related to the presented content and are shareholders of PolTREG S.A. company.

The remaining authors declare that the research was conducted in the absence of any commercial or financial relationships that could be construed as a potential conflict of interest.

## Publisher’s Note

All claims expressed in this article are solely those of the authors and do not necessarily represent those of their affiliated organizations, or those of the publisher, the editors and the reviewers. Any product that may be evaluated in this article, or claim that may be made by its manufacturer, is not guaranteed or endorsed by the publisher.
